# Sigma-1 Receptor Activation Is Protective against TGFβ2-Induced Extracellular Matrix Changes in Human Trabecular Meshwork Cells

**DOI:** 10.3390/life13071581

**Published:** 2023-07-19

**Authors:** Minh Ngoc Tran, Timea Medveczki, Balazs Besztercei, Gyorgy Torok, Attila J. Szabo, Xavier Gasull, Illes Kovacs, Andrea Fekete, Judit Hodrea

**Affiliations:** 1 MTA-SE Lendület “Momentum” Diabetes Research Group, Semmelweis University, 1083 Budapest, Hungary; ngocminhdt@gmail.com (M.N.T.);; 2 Semmelweis University Pediatric Center, MTA Center of Excellence, 1083 Budapest, Hungary; 3 Department of Biochemistry, University of Medicine and Pharmacy at Ho Chi Minh City, Ho Chi Minh City 72712, Vietnam; 4 Institute of Clinical Experimental Research, Semmelweis University, 1094 Budapest, Hungary; 5 Department of Biophysics and Radiation Biology, Semmelweis University, 1094 Budapest, Hungary; 6 Department of Biomedicine, Institute of Neurosciences, University of Barcelona, 08035 Barcelona, Spain; 7 Department of Ophthalmology, Semmelweis University, 1085 Budapest, Hungary; 8 Department of Ophthalmology, Weill Cornell Medical College, New York, NY 10021, USA

**Keywords:** Sigma-1 receptor, fluvoxamine, TGFβ2, trabecular meshwork, extracellular matrix, cytoskeletal remodeling, proliferation, outflow pathway resistance, intraocular pressure, glaucoma

## Abstract

The trabecular meshwork (TM) route is the principal outflow egress of the aqueous humor. Actin cytoskeletal remodeling in the TM and extracellular matrix (ECM) deposition increase TM stiffness, outflow resistance, and elevate intraocular pressure (IOP). These alterations are strongly linked to transforming growth factor-β2 (TGFβ2), a known profibrotic cytokine that is markedly elevated in the aqueous humor of glaucomatous eyes. Sigma-1 receptor (S1R) has been shown to have neuroprotective effects in the retina, but data are lacking about its role in the TM. In this study, we identified the presence of S1R in mouse TM tissue and investigated the effect of an S1R agonist fluvoxamine (FLU) on TGFβ2-induced human TM cells regarding cell proliferation; ECM-related functions, including F-actin reorganization; and the accumulation of ECM elements. TGFβ2 increased the proliferation, cytoskeletal remodeling, and protein levels of fibronectin, collagen type IV, and connective tissue growth factor, and decreased the level of matrix metalloproteinase-2. Most importantly, FLU reversed all these effects of TGFβ2, suggesting that S1R agonists could be potential candidates for preserving TM function and thus maintaining normal IOP.

## 1. Introduction

The trabecular meshwork (TM) located in the iridocorneal angle of the eye is the main drainage pathway of aqueous humor (AH). Structural and functional impairment of the TM is associated with increased AH outflow resistance and intraocular pressure (IOP) elevation [1,2,3,4,5], which are the main risk factors of glaucoma [6,7]. Transforming growth factor-beta2 (TGFβ2) is the predominant isoform of TGFβ in the eye [8,9] and it is known to be strongly associated with the development of elevated IOP and primary open-angle glaucoma [10,11,12]. 

Numerous studies have shown that TGFβ2 increases the secretion and deposition of extracellular matrix (ECM) proteins [10,13,14,15] and connective tissue growth factor (CTGF), which is a downstream mediator of TGFβ2 [13,16,17,18] and a key inducer of ECM synthesis. There is increased deposition of these elements in the TM of glaucomatous eyes, among which fibronectin and collagen are the major components [10,19,20,21,22,23]. Research efforts have shed some light on the complex process of modulating ECM turnover in the TM, but treatment of TM dysfunction is still an unmet need.

The Sigma-1 receptor (S1R) is a multifunctional molecular chaperone, with a well-known protective effect in the central nervous system [24,25,26,27,28]. S1R has also been identified in the eye, including retinal tissue, the iris–ciliary body, lacrimal glands, the cornea, and the lens [29,30,31,32,33]. Studies have already reported its protective effect in the retina [34,35,36,37], but there are no data about S1R abundance and function in the TM except one investigation on pressure-induced cell death [29]. 

Here, we provide evidence about the presence of S1R and about the protective effect of S1R agonist fluvoxamine (FLU) against TGFβ2-induced ECM-related pathologies, including cell proliferation, morphological changes, F-actin enhancement, and rearrangement in the TM. 

## 2. Materials and Methods

### 2.1. Materials

Unless stated otherwise, all standard plastic materials were supplied by Sarstedt (Numbrecht, Germany), all chemical agents were bought from Sigma-Aldrich/Merck (St. Luis, MO, USA), and each experiment was performed at least three times.

### 2.2. Human Trabecular Meshwork Cell Line

Nonglaucomatous transformed human trabecular meshwork cells (HTM5) were developed by Dr. Abbot Clark (University of North Texas—Health Science Center, Denton, TX, USA) [38] and provided by Dr. Xavier Gasull (University of Barcelona) for these experiments. HTM5 cells were grown in culture flasks in Dulbecco’s Modified Eagle Medium (DMEM, 31885-023, Gibco, Thermo Fischer, Waltham, MA, USA) complemented with 1% penicillin/streptomycin (Pen Strep, 15140-122, Gibco) and 10% fetal bovine serum (FBS, 10500-064, Gibco) in an atmosphere containing 5% CO_2_ and 95% air at 37 °C. The cells were passaged every 2–3 days using 0.25% Trypsin-EDTA buffer (25200-072, Gibco). For the characterization of TM cells, the induction of myocilin and alpha smooth muscle actin (α-SMA) was confirmed after 7 days of 100 nM dexamethasone (1177-87-3, Sigma-Aldrich) treatment. The expression of myocilin and α-SMA was investigated using immunolabeling and Western blotting. 

### 2.3. Exposure to TGFβ2 and Fluvoxamine

HTM5 cells were seeded to subconfluence in serum-free DMEM for 24 h. For a dose curve, the cells were activated with various concentrations (1, 2.5, 5, 10, and 20 ng/mL) of TGFβ2 for a period of 24 h iewn serum-free media. To examine the viability of HTM5 cells in response to FLU, we treated the cells with different concentrations (5, 10, and 15 μM) of FLU for 24 h in serum-free media. For the rest of the experiments, cells were induced with 10 ng/mL TGFβ2 alone (302-B2, R&D Systems, Minneapolis, MN, USA) and treated with 10 µM FLU (F2802, Sigma-Aldrich) for 24 h in serum-free media. For each experiment, untreated cells were used as controls.

### 2.4. Proliferation and Cytotoxicity Assay

HTM5 cells were plated in 96-well plates (40,000 cells/100 µL/well, n = 6/treatment group) and treated as mentioned above (Section 2.3). To investigate cell proliferation, after 24 h of various treatments, cells were incubated with methyl-thiazolyldiphenyl-tetrazolium bromide (MTT, 0.5 mg/mL, M6494, Invitrogen) for 3 h at 37 °C. Then, MTT was discarded, and 100 µL of solubilizer (dimethyl sulfoxide (DMSO, D4540, Sigma-Aldrich) was mixed in a 1:1 ratio with ethanol) was added to each well to dissolve the water-insoluble formazan crystals. The absorbance of the formazan solution was recorded at 570 nm (SpectroStar Nano microplate reader, BMG Labtech, Ortenberg, Germany). To assess cell toxicity, the widely used lactate dehydrogenase (LDH) assay (C20300, Invitrogen, Carlsbad, CA, USA) was performed from the supernatant of the cells according to the manufacturer’s protocol and the samples were measured in the same way as in the case of MTT.

### 2.5. Immunocytochemistry

HTM5 cells were plated on gelatin-coated (0.1%, G1393, Sigma-Aldrich) 8-well chamber slides (µ-slide 8-well high Glass Bottom, 80807, Ibidi, Gräfelfing, Germany) (300 µL/well; 150,000 cells/well). After 24 h of treatments (Section 2.3), the cells were washed with phosphate-buffered saline (PBS), followed by fixation with 4% paraformaldehyde (30525-89-4, Sigma-Aldrich) at room temperature for 15 min. Then, the cells were washed, permeabilized with 0.1% TritonX100 (9036-19-5, Sigma-Aldrich) for 10 min, washed again, and blocked with 5% bovine serum albumin (BSA, A2153, Sigma-Aldrich) in PBS for 1 h. Next, repeated washes with PBS were followed by incubation with Alexa 546-phalloidin (1:40, A22283, Invitrogen) for 1 h; then, the cells were washed and the nuclei were counterstained with Hoechst 33342, (1 µg/mL, Invitrogen, Darmstadt, Germany) for 10 min. Finally, after rinsing with PBS, coverslips were mounted onto slides using ProLong antifade (P36980, Invitrogen). Fluorescent images were acquired with a Nikon Eclipse Ti2 inverted microscope (Nikon Instruments, Melville, NY, USA) equipped with 10×, 20×, 40×, and 60× oil immersion objective (Plan Apo lambda, N.A. 1.4) plus a 1.5× intermediate magnification and a cooled sCMOS camera (Zyla 4.2, Andor Technology, Belfast, UK). Image analysis for F-actin quantification was performed on images captured with 10× objective using NIS-Elements (Nis Elements 5.21.03 software version, Nikon Instruments) and data are presented as integrated density. To visualize the F-actin filaments, 60× objective plus 1.5× intermediate magnification was used and z-stack images were captured. Deconvolution was performed on the cropped 512 × 512-pixel cropped images using Huygens Essential 21.10 (Scientific Volume Imaging, Hilversum, The Netherlands) [39].

### 2.6. Western Blot Analysis

HTM5 cells were seeded at 100,000 cells/mL in 12-well plates (1 mL/well) using DMEM supplemented with 10% FBS. After 24 h, the nutritious media were replaced with serum-free DMEM for an additional 24 h, which was then followed by treatments (Section 2.3) for another 24 h. At the end of treatment, whole-cell lysates were collected. The cells were rinsed with ice-cold PBS and lysed in a lysis buffer containing 1.7 mg/mL aprotinin, 1 M Tris base, 5 mg/mL leupeptin, 0.5 M EGTA, 0.25 M NaF, 1% TritonX100, 0.5 M PMSF, and 0.5 M Na_3_VO_4_. Then, the cells were vortexed for 1 min, sonicated 2 times for 3 min, and vortexed for 30 s, followed by 1 min cooling on ice after each step. The lysates were then cleared using centrifugation at a speed of 13,000 rpm at 4 °C for 10 min. The supernatant containing proteins was transferred to a clean Eppendorf tube and the protein concentration was measured using a Lowry assay with a Protein DC kit (5000113-115, Bio-Rad Laboratories, Inc., Hercules, CA, USA). Then, 4× Laemmli sample buffer (161-0747, Bio-Rad) supplemented with 2-mercaptoethanol (M3148, Sigma-Aldrich) was added to the samples, which were all denatured by heating at 95 °C for 5 min. In the case of collagen type IV, we used 4X Laemmli buffer without a reducing agent and the denaturing step was omitted according to the manufacturer’s protocol. Total protein (10 µg) was loaded to polyacrylamide gradient gels (4–20%) (4561096, Bio-Rad) and transferred to nitrocellulose membranes (1704158, Bio-Rad). The membranes were stained in Ponceau S (0.1%, P3504, Sigma-Aldrich) for 3 min to correct for any differences in total protein loading. After destaining Ponceau S with distilled water, the membranes were blocked by 5% nonfat dry milk or 5% BSA in Tris-buffered saline at room temperature for 1 h and then incubated with primary antibodies (S1R, 1:1000, Santa Cruz sc137075; fibronectin, 1:5000, Abcam ab2413; collagen type IV, 1:500, Abcam ab6586; CTGF, 1:200, Santa Cruz sc14939; MMP2, 1:2000, Abcam ab92536) at 4 °C overnight. The membranes were washed and then probed for secondary antibodies (1:3000, Cell Signaling, Danvers, MA, USA). The signals were detected using a ChemiDoc^TM^ MP Imaging System (Bio-Rad) and analyzed using Image Lab software (version 6.1.0, Bio-Rad).

### 2.7. In Vivo Experiments

#### 2.7.1. TGFβ2 Injection

Four-month-old male C57BL/6J mice were purchased from Animalab (Budapest, Hungary) and were kept in a 12 h light/12 h dark cycle at constant temperature (22 ± 2 °C) with ad libitum access to food and water. The animal studies were carried out in accordance with the Animal Experiment Regulations of the Animal Welfare Committee of Semmelweis University (PE/EA/916-7/2020), Budapest, Hungary. 

3.5 µL of TGFβ2 (5 ng/µL) or PBS was injected intracamerally twice a week using a Hamilton glass microsyringe (5 mL volume, Hamilton Company, Reno, NV, USA). For the injection, the mice were anesthetized with 90 mg/10 mg/bwkg of ketamine/xylazine. In addition, 0.4% oxybuprocainhydrochloride (Novesine, OmniVision GmbH, Puchheim, Germany) was used as topical anesthesia. After 5 injections, the eyes were enucleated and processed for further investigations.

#### 2.7.2. Fluorescent Immunohistochemistry for Mouse Anterior Segments

After enucleation, the eyes were fixed in 4% paraformaldehyde overnight at 4 °C and then kept in PBS with 0.01% sodium azide at 4 °C. First, the eyes were dissected and only the anterior segments (ASs) were used for the staining. The ASs were washed with PBS 4 times and then permeabilized with 0.5% TritonX100 for 1 h at room temperature, washed again, and blocked with 5% goat serum (31873, Invitrogen), 1% BSA, and 0.1% TritonX100 in PBS for 1 h at room temperature. Next, the ASs were incubated with the primary antibody (S1R, 1:50, Invitrogen, 42-3300) at 4 °C overnight. Then, repeated washes with PBS were followed by incubation with the secondary antibody (goat anti-rabbit Star Red, 1:500, Abberior, Star Red-1002) combined with Hoechst (5 µg/mL) for 1 h. After being washed with PBS, the ASs were stained for F-actin using Phalloidin Star Orange (Abberior, 1:20, Star Orange-0100) and for staining the nuclei, Hoechst was used (5 µg/mL). Both were applied for 1 h at room temperature. Finally, after rinsing with PBS, the samples were kept at 4 °C until further use. Fluorescent images were acquired with an Abberior Expert Line confocal microscope (Abberior, Instruments, Göttingen, Germany).

### 2.8. Statistical Analysis

The data were analyzed statistically using Prism software (version 8.0, GraphPad, Boston, MA, USA). Normal (Gaussian) distribution was checked using a Shapiro–Wilk normality test. Multiple comparisons and interactions were evaluated with a one-way ANOVA followed by a Holm–Sidak post hoc test. For nonparametric data, the Kruskal–Wallis ANOVA on ranks followed by Dunn’s correction was used. All data are presented as mean ± SEM and *p* < 0.05 was considered significant.

## 3. Results

### 3.1. S1R Is Present in Mouse TM and in HTM5 Cells, TGFβ2 Elevates F-Actin Level in Mouse Anterior Segments and Does Not Affect the Level of S1R

**Figure 1:**
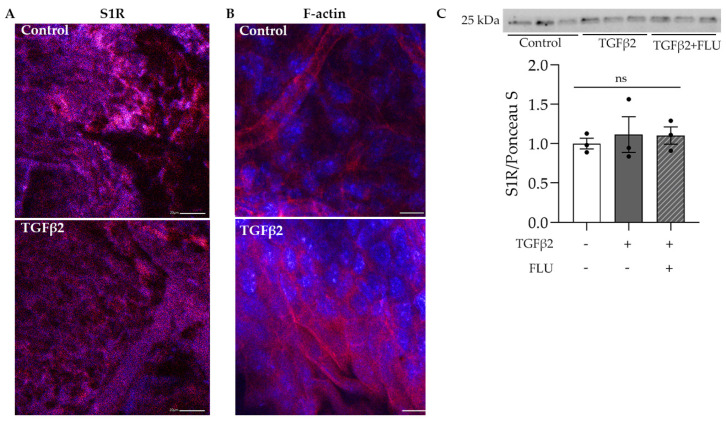
Confocal microscope images of the mouse anterior segments and Western blot of HTM5 cells. (**A**) Sigma-1 receptor (S1R) expression in the trabecular meshwork region of C57BL/6J mice in control and TGFβ2-injected mice (S1R: red, nuclei: blue, 20× objective, scale bar = 20 µm). (**B**) F-actin level in the anterior segment of TGFβ2-injected mice compared to control mice (F-actin: red, nuclei: blue, 40× objective, scale bar = 10 µm). (**C**) Representative Western blot of S1R (25 kDa) protein in HTM5 cells. Cells were treated with 10 ng/mL TGFβ2 alone or in combination with 10 µM FLU for 24 h (data: mean ± SEM; n = 3/group; ns: nonsignificant; ANOVA followed by Holm–Šidak multiple comparison test). The uncropped blots are shown in File S1.

The protective role of S1R in the retina has been widely investigated [34,35,36,37]; however, much less is known about its function in the anterior segment, especially in the TM tissue. In the first step, we visualized S1R in the mouse anterior segments. Confocal immunofluorescence microscope images confirmed the abundant presence of S1R in the TM region, which was not altered by the profibrotic TGFβ2 (Figure 1A). Additionally, we demonstrated the presence of S1R in HTM5 cells. Moreover, Western blot analysis revealed that TGFβ2 and FLU treatment did not change the level of S1R protein (Figure 1C).

The normal function and structure of this spongy TM tissue are crucial in maintaining IOP and changes in its ECM have been associated with POAG [1,2,3,4,5]. In addition, TGFβ2, which is the main isoform of TGFβ, has been found in increased amounts in the aqueous humor of POAG patients [11,40] and proved to be involved in the ECM production and degradation of TM [10,13,14,15]. To verify the effect of TGFβ2 in the TM, C57BL/6J mice were injected with TGFβ2, and the anterior segments were stained for F-actin, an essential component of the ECM. Fluorescent confocal microscopy images revealed a massive increase in the F-actin level upon TGFβ2 induction in the TM region (Figure 1B), a result that indicated the usage of TGFβ2 as an ECM-altering agent in our further investigations.

Secondly, we aimed to elucidate the role of S1R in ECM-related changes in the TM; the direct effect of the specific S1R agonist FLU on TGFβ2-induced human trabecular meshwork cell culture (HTM5) was investigated.

### 3.2. FLU Prevents TGFβ2-Induced Cell Proliferation and Morphological Changes and Is Not Toxic to the Cells

Before performing experiments, to confirm the characteristics of HTM5 cells, dexamethasone treatment was applied as previously described [41], and the induction of myocilin and αSMA was detected with immunocytochemistry (Supplementary Materials).

**Figure 2:**
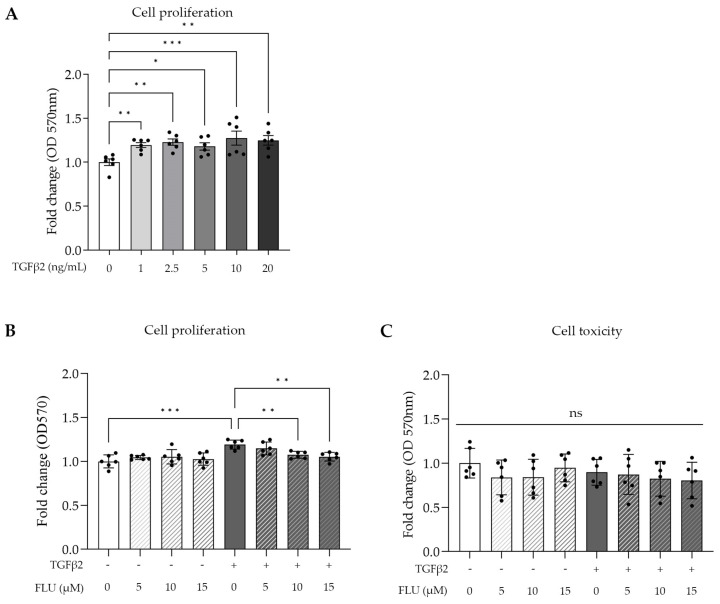
Cell proliferation and cell toxicity of human trabecular meshwork (HTM5) cells after TGFβ2 stimuli and fluvoxamine (FLU) treatment. (**A**) Cell proliferation after treatment with different concentrations of TGFβ2 (0, 1, 2.5, 5, 10, and 20 ng/mL; 24 h). (**B**) Cell proliferation treatment with 10 ng/mL TGFβ2 combined with 5, 10, or 15 µM FLU (24 h). (**C**) Cellular toxicity determined with LDH assay after 24 h induction with 10 ng/mL TGFβ2 and/or treatment with FLU (5, 10, and 15 µM). (Data: mean ± SEM. * *p* < 0.05, ** *p* < 0.01, *** *p* < 0.001, ns: nonsignificant; n = 6/group; one-way ANOVA.)

TGFβ2 is involved in cell proliferation; therefore, we determined its lowest concentration needed for a maximum effect on TM cell proliferation, using an MTT assay, which is a common colorimetric method to measure proliferation and cell viability in vitro. The HTM5 cells were activated with TGFβ2 in a range of 1–20 ng/mL for 24 h. Dose-dependent cell growth was measured with a peak at 10 ng/mL (Figure 2A).

Based on this, 10 ng/mL TGFβ2 was used in further experiments. To assess the impact of FLU on cell proliferation and cellular toxicity, an MTT assay and a commonly used lactate dehydrogenase assay (LDH) were performed, respectively. The cells were treated with three different concentrations of FLU (5, 10, and 15 µM; 24 h), which were applied to the cells alone or in combination with TGFβ2. The MTT results revealed that FLU prevented TGFβ2-induced cell proliferation in a dose-dependent manner. However, when FLU was applied alone, it did not affect the proliferation (Figure 2B). Based on these findings, for our subsequent experiments, we selected the minimum effective dose of FLU required for proliferation inhibition, which in this case was determined to be 10 μM.

The cytotoxicity measurements confirmed our expectations, as none of the treatments exhibited cytotoxicity. This included FLU alone at concentrations of 5, 10, or 15 µM; TGFβ2 at a concentration of 10 ng/mL; and the combination of TGFβ2 with FLU at concentrations of 5, 10, or 15 µM (Figure 2C). The cells remained unaffected by these treatments, indicating their nontoxic nature.

**Figure 3:**
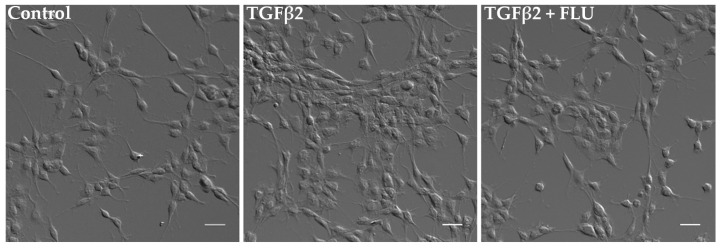
Morphology of human trabecular meshwork (HTM5) cells. Cells were treated with 10 ng/mL TGFβ2 alone or in combination with 10 µM FLU for 24 h (20× objective; scale bar = 20 µm).

In parallel, the cells’ morphological changes were visualized. Consistent with the MTT results, differential interference contrast (DIC) images confirmed a noticeable increase in cell proliferation upon TGFβ2 induction. However, the presence of FLU ameliorated this effect, as observed in the images (Figure 3). 

### 3.3. FLU Attenuates TGFβ2-Induced Cytoskeletal Remodeling

**Figure 4:**
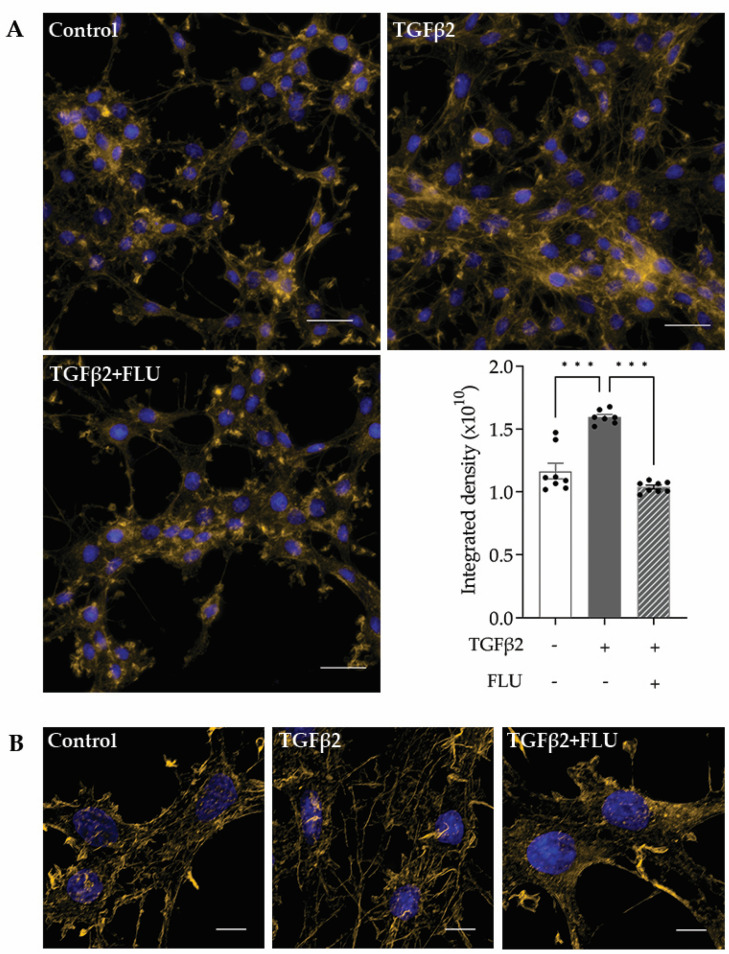
F-actin reorganization in HTM5 cells. Representative images of cytoskeletal rearrangement after 10 ng/mL TGFβ2 induction for 24 h with or without 10 µM FLU with (**A**) smaller and (**B**) higher magnification. F-actin was visualized with phalloidin-Alexa Fluor 546 (F-actin: yellow, nuclei: blue; (**A**) 60× objective along with a 1.5× intermediate magnification, scale bar = 20 µm. Integrated density values represent quantification of fluorescence (data: mean ± SEM. *** *p* < 0.001; n = 6–8/group; one-way ANOVA). (**B**) 512 × 512-pixel cropped deconvolved images (60× objective along with a 1.5× intermediate magnification, scale bar = 5 µm).

F-actin, or filamentous actin, plays a critical role in many cellular processes, including cell movement and the maintenance of cell shape [42,43]. In the TM, the F-actin network maintains the ECM surrounding the trabecular meshwork cells, and that plays a role in modulating the AH outflow facility. Therefore, investigating the effect of FLU on the actin cytoskeleton is rational. The representative fluorescent images of phalloidin-stained HTM5 cells showed a marked induction of F-actin level upon TGFβ2 induction, with increased actin clump and stress fiber formation, compared to the diffuse actin network of thin actin filaments seen in the control cells (Figure 4A). 

The S1R agonist FLU reduced F-actin enhancement; furthermore, fewer stress fibers and less clump accumulation were observed. To clearly visualize the actin stress fibers, image deconvolution was performed on the cropped images using Huygens Deconvolution software, Huygens Essential 21.10 (Figure 4B). This postprocessing step enables the resolution of finer details in the image. In addition to visualization, a quantification of the fluorescent signal was performed, and it is presented as integrated density, which is a commonly used metric in image evaluation (Figure 4A, lower panel).

All these data confirmed the protective effect of FLU on TGFβ2-induced F-actin network reorganization in HTM5 cells. 

### 3.4. FLU Ameliorates the TGFβ2-Induced Production of ECM Compounds

There is an increased deposition of ECM elements in the TM of glaucomatous eyes, among which fibronectin and collagen are the main components [10,11,19,20,21,22,23]. In addition to this, CTGF stimulates the accumulation and remodeling of the ECM [16,18,44]. 

**Figure 5:**
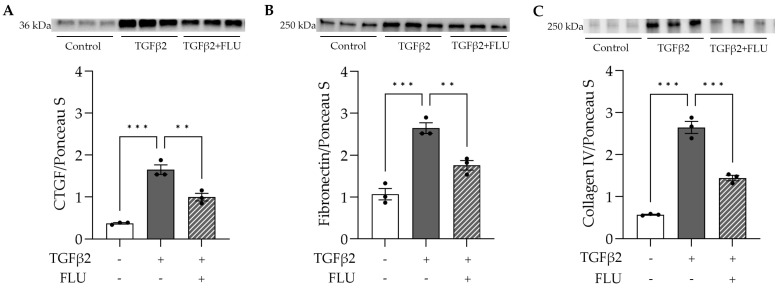
ECM-related protein levels in HTM5 cells after induction with 10 ng/mL TGFβ2 for 24 h alone or in combination with 10 µM FLU. (**A**) Representative Western blot images of CTGF (36 kDa), (**B**) fibronectin (250 kDa), and (**C**) collagen IV (250 kDa) (all data: mean ± SEM. ** *p* < 0.01, *** *p* < 0.001; n = 3/group; one-way ANOVA). The uncropped blots are shown in File S1.

To assess the effect of FLU on the ECM’s key players, HTM5 cells were treated with TGFβ2 and FLU, and CTGF, fibronectin, and collagen type IV were analyzed with a Western blot. In line with the literature, in the HTM5 cells, TGFβ2 markedly increased the levels of CTGF, fibronectin, and collagen IV, while FLU suppressed the elevation of all proteins (Figure 5). To the best of our knowledge, these results are the first evidence for the impact of FLU on these ECM components in TM cells. 

### 3.5. FLU Increases the Level of the ECM-Degrading Enzyme MMP2

**Figure 6:**
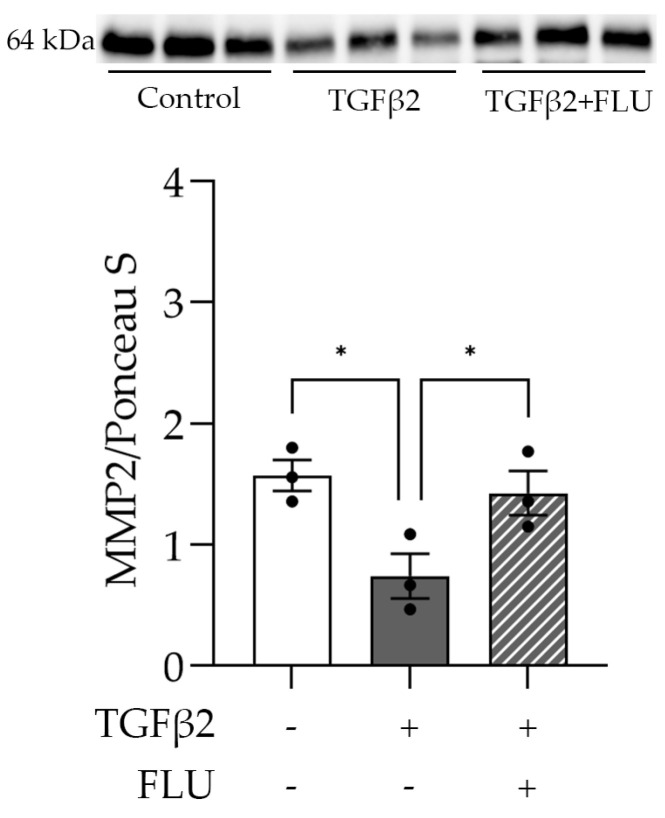
Level of matrix metalloproteinase-2 (MMP2) in HTM5 cells after induction with 10 ng/mL TGFβ2 for 24 h alone or in combination with 10 µM FLU. Western blot of ECM-degrading MMP2 enzyme (64 kDa) (Data: mean ± SEM. * *p* < 0.05; n = 3/group; one-way ANOVA). The uncropped blots are shown in File S1.

One may speculate that FLU attenuates ECM accumulation by facilitating its degradation. To verify this, we examined the level of the active form of the proteolytic enzyme MMP2 that plays a role in the breakdown of ECM components. As the Western blot analysis revealed, TGFβ2 reduced the MMP2 level (Figure 6). More importantly, the TGFβ2-induced suppression of MMP2 was reversed by FLU (Figure 6), and this may facilitate ECM degradation. This is in line with our F-actin and ECM element measurements and confirms the protective effect of FLU against TGFβ2-induced alterations in HTM5 cells. 

## 4. Discussion

S1R is a ubiquitously expressed chaperone that has been studied rigorously in the brain, with a large body of evidence supporting its neuroprotective effect in various central nervous system diseases [24,25,26,27,28,45]. S1R is located in various ocular tissues; however, most papers have reported on its retinal effects [34,35,36,37,46,47,48,49]. There is only one study, which investigated the role of S1R in the pathologies of TM, showing that S1R agonist (+)-pentazocine protects human TM cells from pressure-induced apoptosis [29]. Our study is the first that deeply investigates S1R abundance and function in the anterior segment of the eye (more precisely in the TM). Additionally, we confirmed the presence of S1R in HTM5 cells. Moreover, we demonstrated that none of the treatments altered the protein level of S1R.

TGFβ2 is the predominant isoform of TGFβ in the eye and is one of the two factors (along with dexamethasone) that is known to cause glaucomatous alterations in the TM. Although numerous investigations have been performed to examine TGFβ2’s effect on ECM remodeling and TM function [10,11,12,13,14,15], the detailed mechanism leading to increased AH outflow resistance and elevated IOP is still not clear. Novel compounds that could ameliorate TM dysfunctions are of immense interest. 

Here, we provide evidence that the S1R agonist FLU prevents ECM-related alterations of HTM5 cells caused by TGFβ2. In line with the literature, we also detected F-actin enhancement and the formation of stress fibers and actin clumps upon TGFβ2 induction both in vivo (Figure 1B) and in vitro (Figure 4). Similarly to other studies [14,15,19,20], we confirmed that TGFβ2 significantly increased cell proliferation (Figure 2), caused morphological changes (Figure 3), and induced F-actin level and rearrangement (Figure 4) and the accumulation of ECM elements (Figure 5) in HTM5 cells. More importantly, we demonstrated the protective effect of FLU by mitigating various changes, including the downregulation of F-actin, CTGF, fibronectin, and collagen IV proteins (Figure 4 and Figure 5), which are key factors in maintaining normal outflow resistance in the TM. Additionally, our study reveals that none of the treatments induced cellular toxicity, affirming the safety of allying FLU.

Multiple possibilities have to be considered beyond this protective effect against TGFβ2-induced changes. First of all, FLU markedly decreased the level of F-actin, the main component of the cell’s cytoskeleton, and inhibited actin clump and stress fiber formation. Thus, cellular processes such as cell division, cell movement, and the maintenance of cell shape, in all of which F-actin plays a critical role [42], are directly targeted by FLU.

Secondly, the effect of FLU on CTGF is also a possibility. CTGF is involved in a variety of biological processes, including cell adhesion and migration. It is well known to stimulate the deposition and remodeling of the ECM [16,17,44], while its inhibition could attenuate fibrotic processes [17,44]. We postulate that the increased fibronectin and collagen IV level could be a consequence of CTGF activation. Therefore, its inhibition by FLU can lead to the decreased level of these fibrotic proteins, and through this, FLU could ameliorate tissue stiffness. However, further research is needed to fully understand their potential interactions. 

Targeting ECM degradation is another manner in which FLU might be protective. The accumulation of the ECM could be the result of the reduction in ECM-degrading enzyme activity (MMPs and some other proteolytic enzymes). The inhibition of MMP2 expression or activity in human TM cell cultures through TGFβ2 treatment has been reported [15,50]. The overexpression of ECM elements (fibronectin and collagen IV) and reduction in the MMP2 trigger fibrotic processes and, therefore, may increase AH outflow resistance. We found that FLU prevented the TGFβ2-induced MMP2 decrease (Figure 6). Therefore, we propose that it could maintain the ECM-degrading function of the enzyme. It is worth noting that further experiments investigating the enzyme activity using gel zymography would provide further details about the mechanism of FLU’s effect on ECM decomposition.

In conclusion, our research describes the presence of S1R in TM cells. We reported a novel effect of the S1R agonist FLU, which protects TM cells from ECM remodeling, accumulation, and dysfunction caused by the TGFβ2/CTGF pathway. Therefore, we postulate that S1R activation is a valuable approach to preserve the TM’s structure and function. Investigating the effect of FLU in vivo is a feasible and rational continuation that will further validate our in vitro results.

## 5. Patents

Patent number: US20190209575A1.

## Data Availability

Not applicable.

## Supplementary Materials

The following supporting information can be downloaded at: https://www.mdpi.com/article/10.3390/life13071581/s1, Figure S1: Immunocytochemistry and Western blot of HTM5 cells after treatment with 100 nM dexamethasone (Dex) for 7 days. File S1: Full pictures of the Western blots.
