# Supplementary material for: Sigma-1 Receptor Activation Is Protective against TGFβ2-Induced Extracellular Matrix Changes in Human Trabecular Meshwork Cells
Source: Life (Basel). 2023 Jul 19;13(7):1581. doi: 10.3390/life13071581 (PMC10381521; doi:10.3390/life13071581)
Supplement: Supplementary file 1 [file life-13-01581-s001.zip › Figure S1.pdf]

Characterization of TM cells

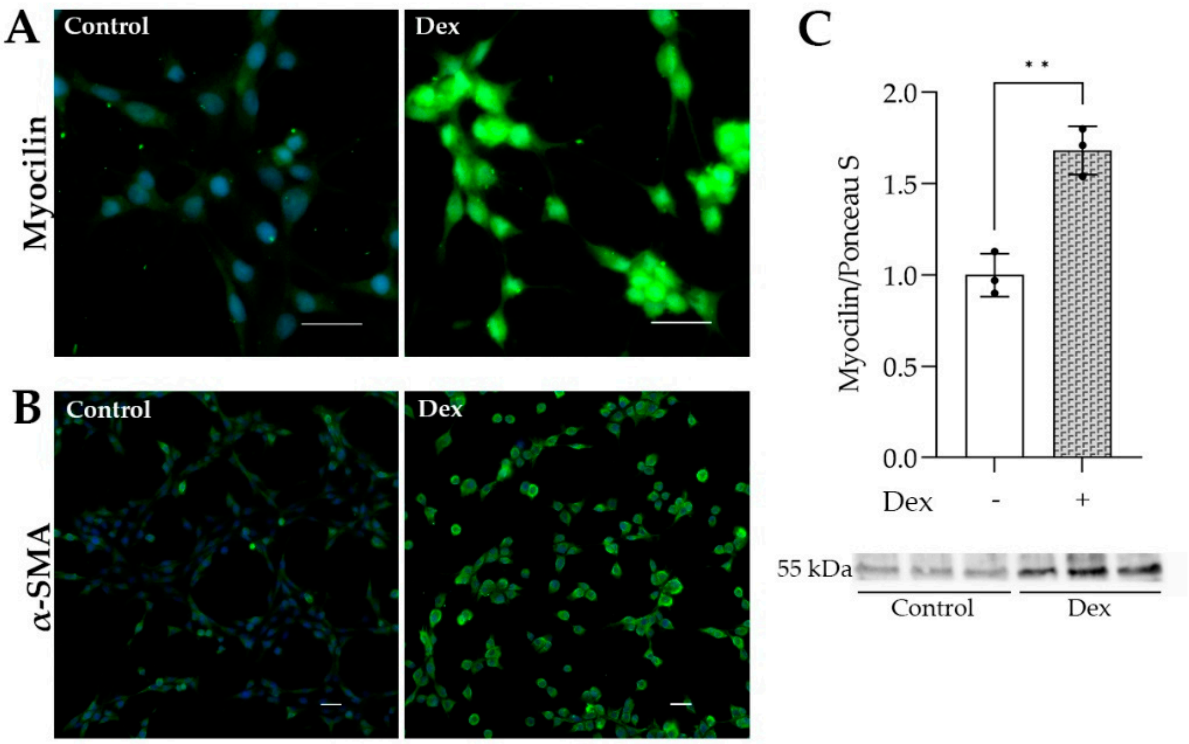

Figure S1. Immunocytochemistry and western blot of HTM5 cells after treatment with 100 nM dexamethasone (Dex) for 7 days. (**A**) Difference of myocilin expression between Dex-treated cells and non-treated cells (myocilin: green, nuclei: blue, 400X magnification, scale bar = 20 μm). (**B**) Alpha smooth muscle actin (αSMA) level in the TM cells treated with Dex compared to control (αSMA: green, nuclei: blue, 400X magnification, scale bar = 20 μm). (**C**) Representative western blot of myocilin (55 kDa). Data were presented as mean ± SEM. **$p<0.01$, n=3/group, unpaired *t-test*.

Dexamethasone increases the expression of myocilin and αSMA of human trabecular meshwork cells.
