# Supplementary material for: Sigma-1 Receptor Activation Is Protective against TGFβ2-Induced Extracellular Matrix Changes in Human Trabecular Meshwork Cells
Source: Life (Basel). 2023 Jul 19;13(7):1581. doi: 10.3390/life13071581 (PMC10381521; doi:10.3390/life13071581)

Ponceau S for Sigma-1 receptor expression

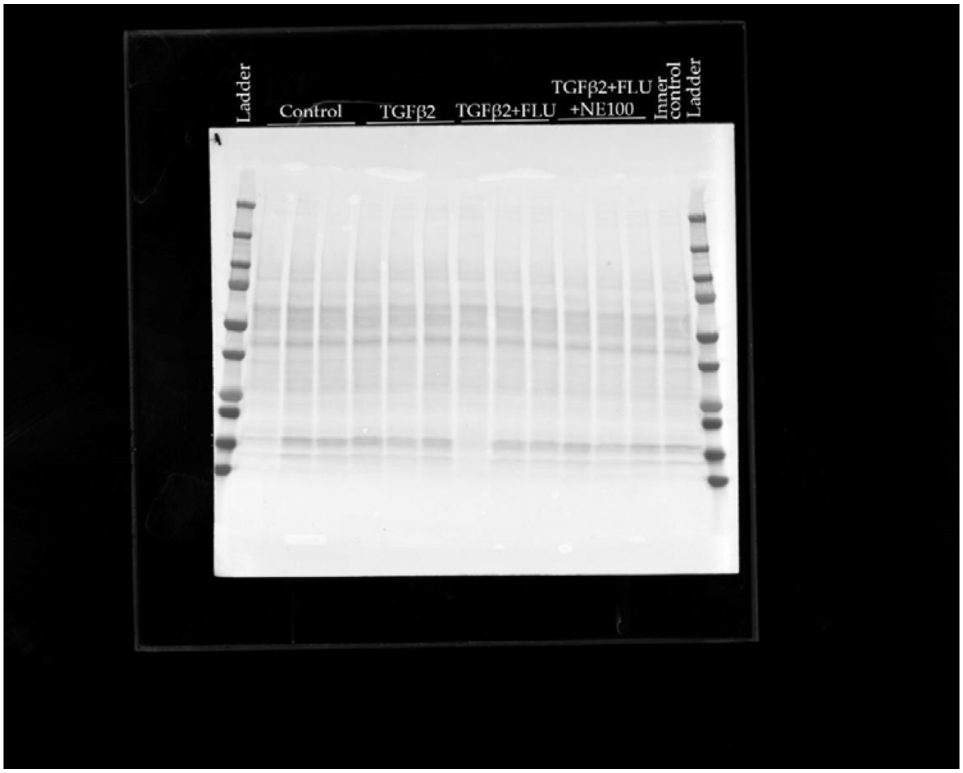

Membrane for Sigma-1 receptor expression (25 kDa)

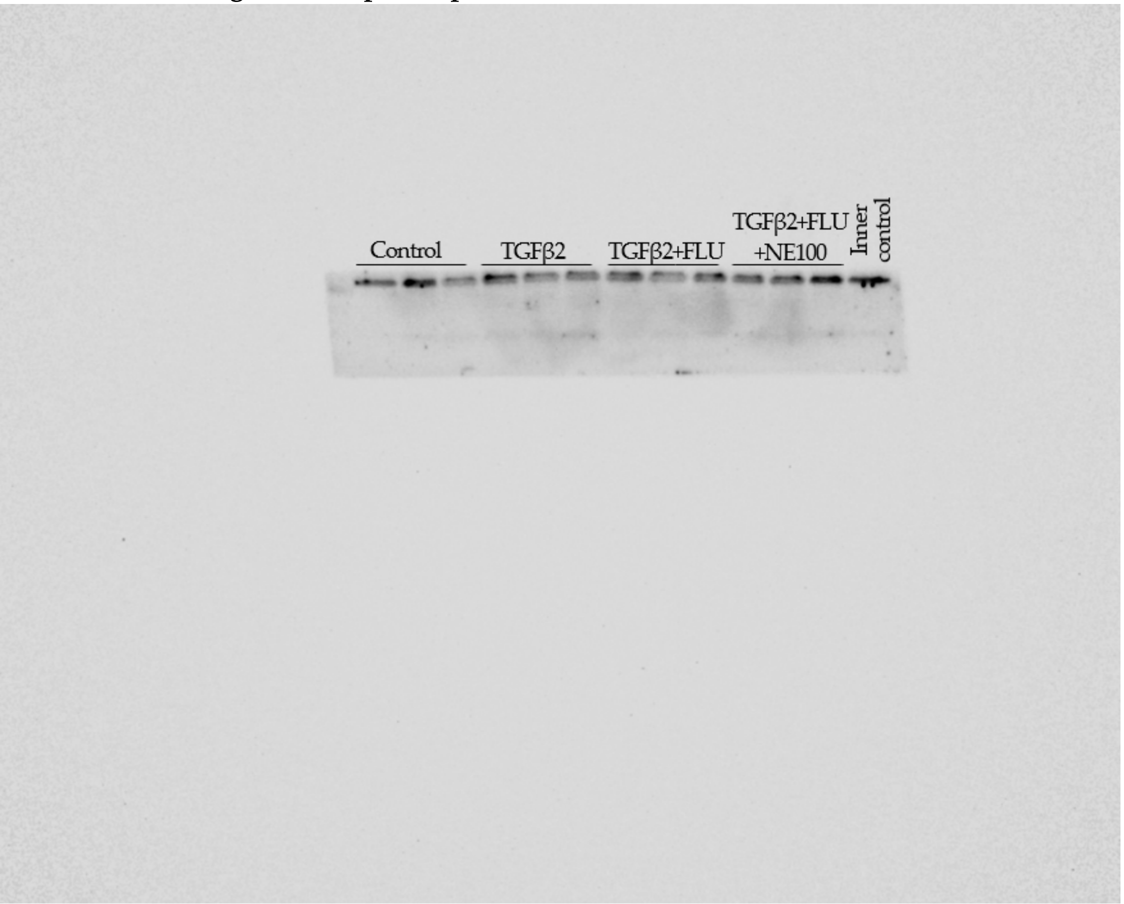

Ponceau S for Myocilin expression

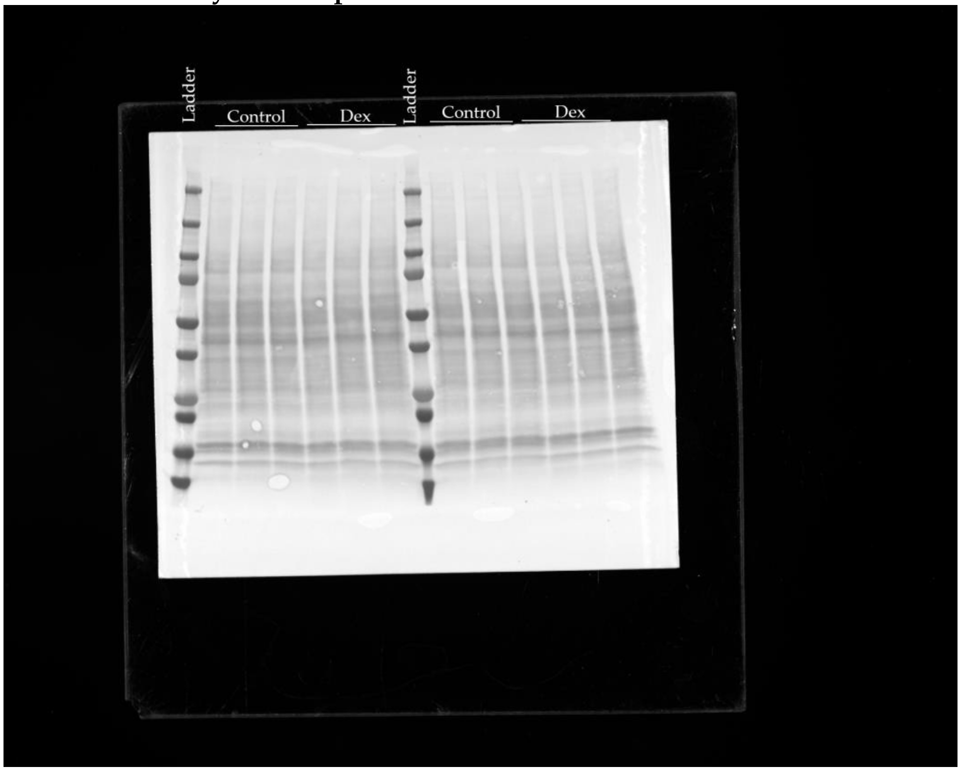

Membrane for Myocilin expression (55 kDa)

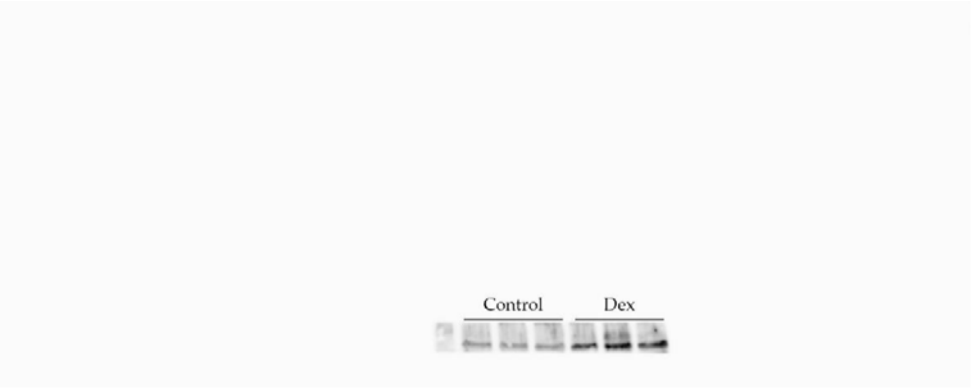

Ponceau S for Fibronectin and CTGF expression

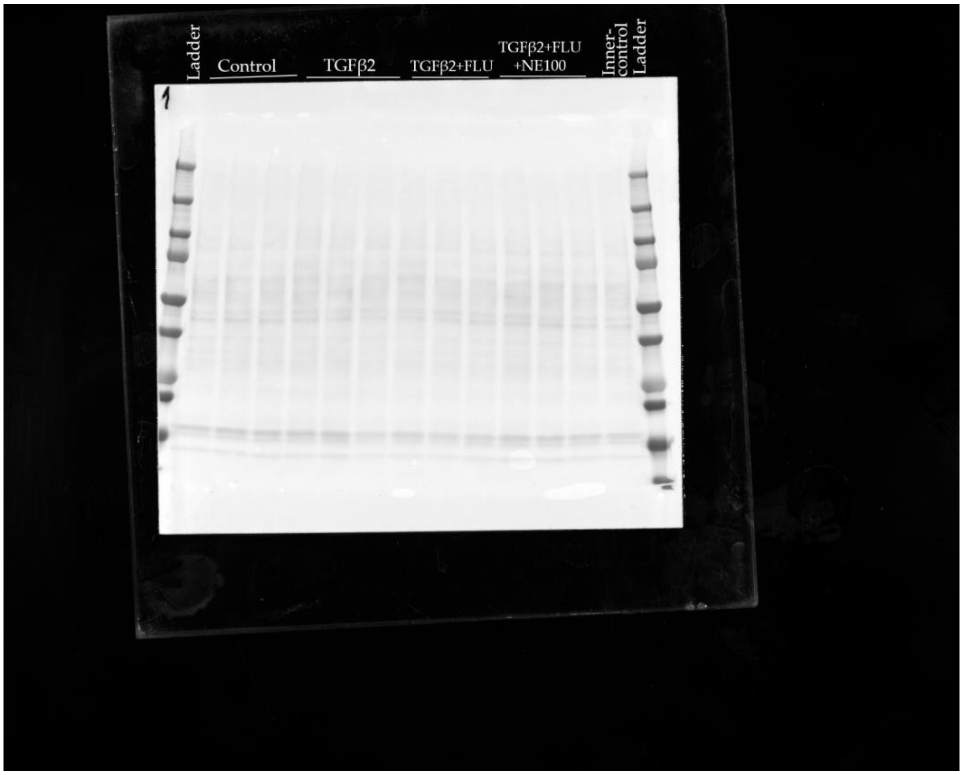

Membrane for Fibronectin expression (250 kDa)

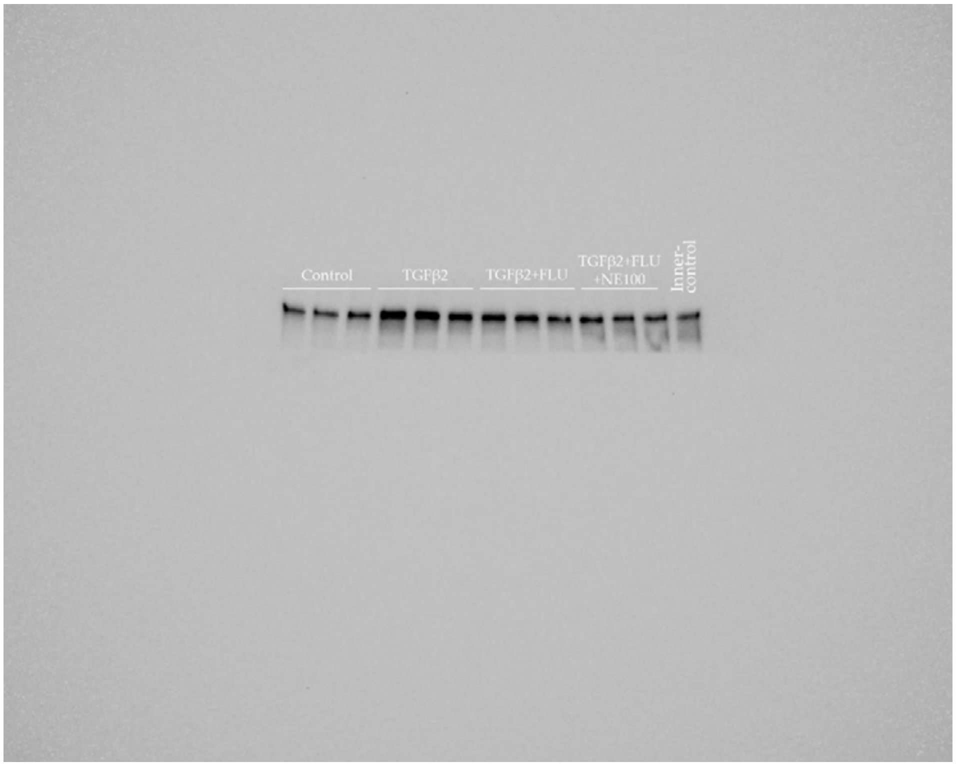

Membrane for CTGF expression (36 kDa)

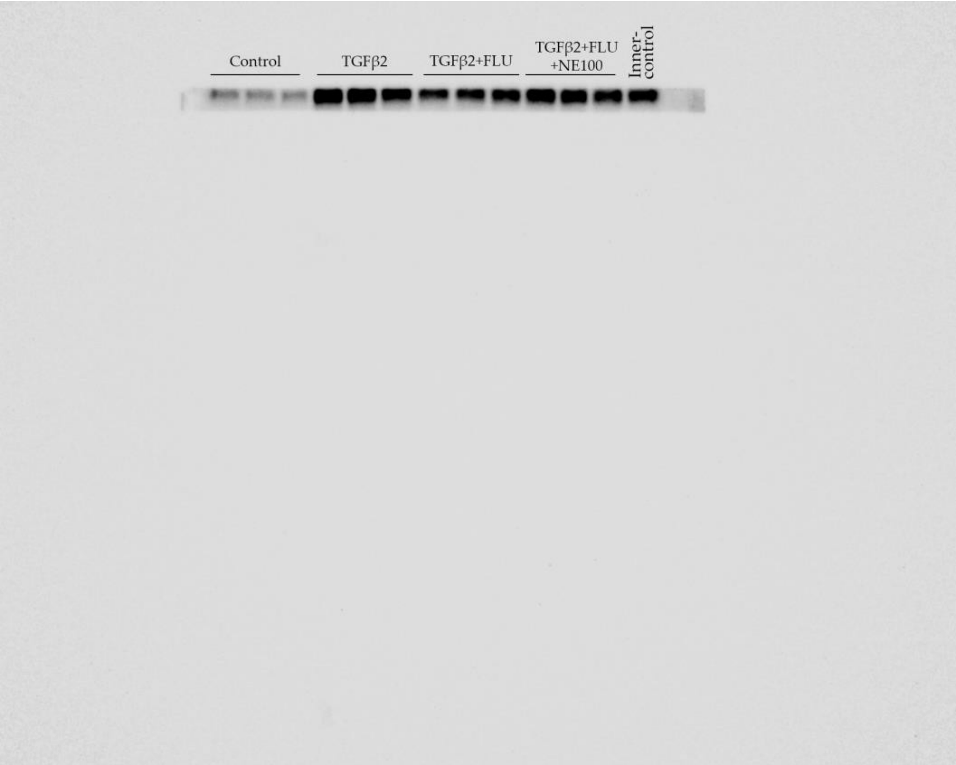

Ponceau S for Collagen type 4 expression

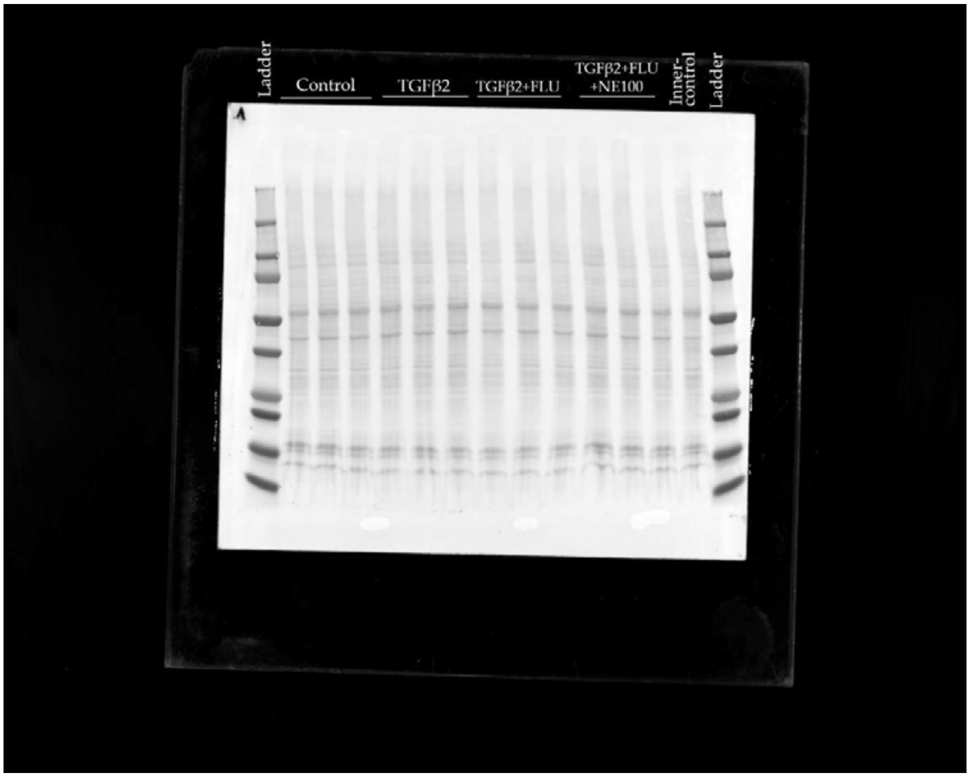

Membrane for Collagen type 4 expression (250 kDa)

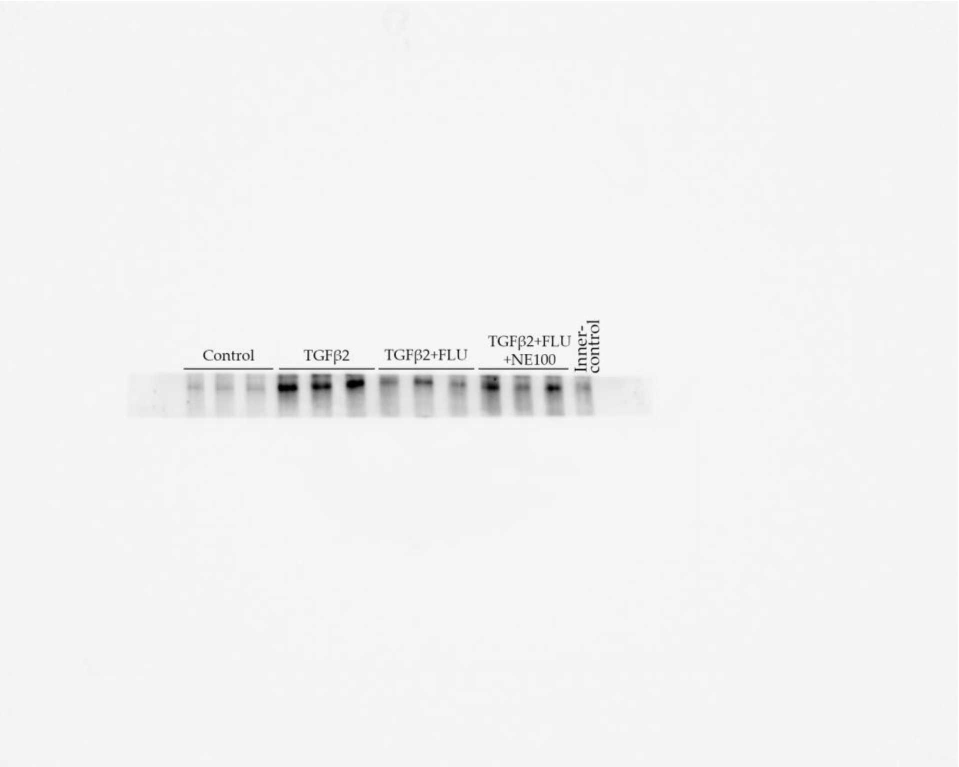

Ponceau S for MMP2 expression

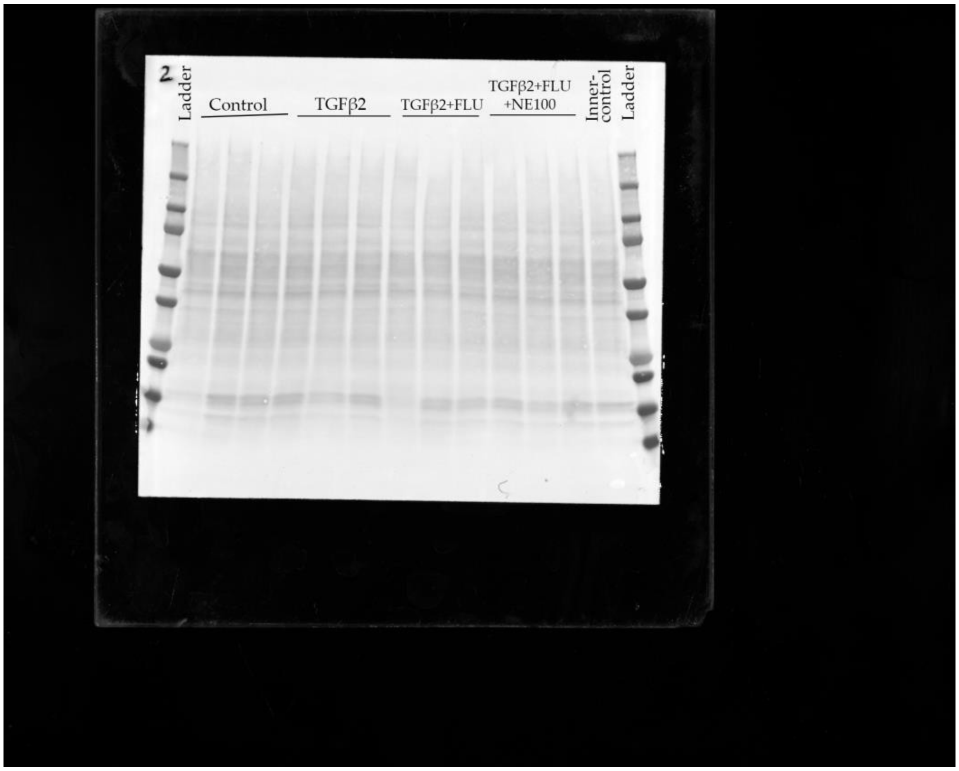

Membrane for MMP2 expression (64 kDa)

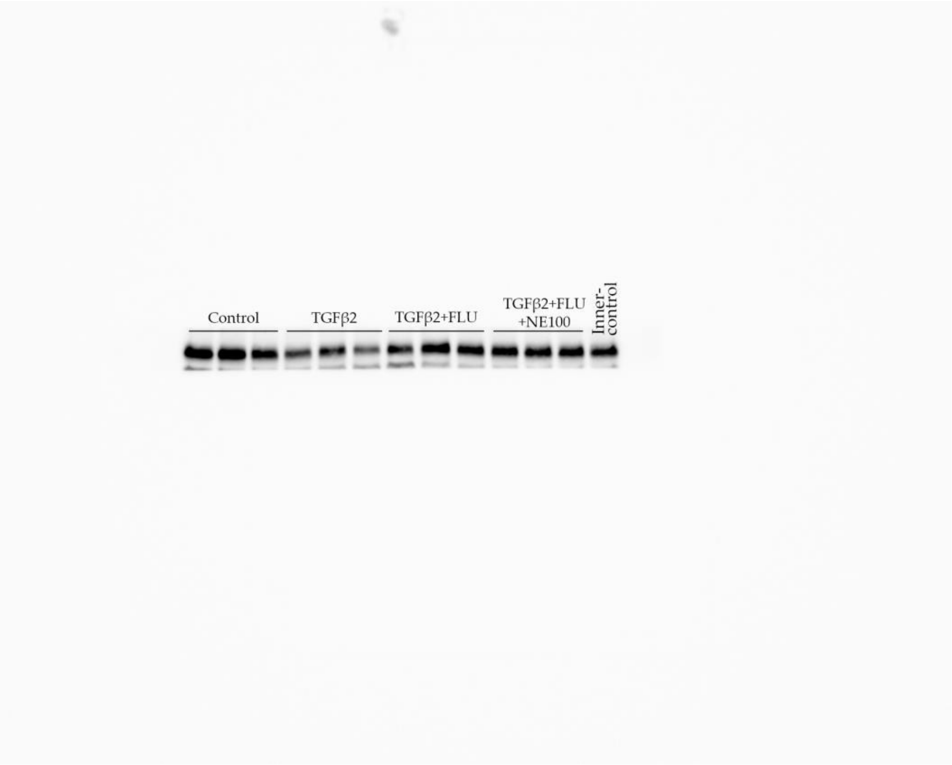

Supplement: Supplementary file 1 [file life-13-01581-s001.zip › File S1.pdf]
